# Child and Adolescent Behavior Inventory (CABI): Standardization for Age 6-17 Years and First Clinical Application

**DOI:** 10.2174/1745017901713010020

**Published:** 2017-03-22

**Authors:** Carlo Cianchetti, Marcello Pasculli, Andrea Pittau, Maria Grazia Campus, Valeria Carta, Roberta Littarru, Giuseppina Sannio Fancello, Alessandro Zuddas, Maria Giuseppina Ledda

**Affiliations:** 1 Child & Adolescent Neurology and Psychiatry Unit, University of Cagliari, Cagliari, Italy; 2 Child & Adolescent Neuropsychiatry Unit, Department of Biomedical Science, University of Cagliari & “A. Cao” Microcitemico Paediatric Hospital – G. Brotzu Hospital Trust, Cagliari, Italy; 3 Gnosis Center for Neuropsychological and Emotional Evaluation, Cagliari, Italy

**Keywords:** CABI, CBCL, Questionnaire, Inventory, Behavior, Internalizing, Externalizing, Screening

## Abstract

**Background:**

The Child and Adolescent Behavior Inventory (CABI) is a questionnaire designed to collect information from the parents of children and adolescents, both for the preparation of screening and epidemiological studies and for clinical evaluation. It has been published in CPEMH in 2013, with the first data on 8-10 years old school children.

Here we report an extended standardization on a school population 6-17 years old and the first results of the application in a clinical sample.

**Methods:**

Parents, after giving their informed consent, answered to the questionnaire. Complete and reliable data were obtained from the parents of 659 school children and adolescents 6-17 y.o., with a balanced distribution of gender.

Moreover, in a population of 84 patients, the results with the CABI were compared with the clinical evaluation and the CBCL.

**Results:**

In the school population, scores were different in relation to gender and age. The values of externalizing disorders were higher in males, with the highest values for ADHD in the 6-10 y.o. children. On the contrary, the scores of internalizing disorders and of eating disorders tended to be slightly higher in females.

In the clinical population, scores at the CABI were in agreement with the clinical evaluation in 84% cases for depressive symptoms (compared to CBCL 66%), 53% for anxiety symptoms (CBCL 42%) and 87% for ODD (CBCL 69%), differences, however; without statistical significance (chi square).

**Conclusion:**

The study obtained normative data for the CABI and gave information of the behavioral differences in relation to age and gender of the school population as evaluated by parents/caregivers. Clinically, the CABI provided useful information for the clinical evaluation of the patient, sometimes with better agreement with the final diagnosis compared to the CBCL.

## INTRODUCTION

The Child and Adolescent Behavior Inventory (CABI) is a questionnaire for parents and caregivers, who are asked to respond to items concerning the behavior of their child or adolescent [1]. It includes 75 items exploring nearly all areas of psychopathology (the whole text is freely available in the Appendix of [1]). Data on the construction and validation of the CABI for 8 to 10 years old children are reported by Cianchetti *et al.* [1].

In the questionnaire, the parent/caregiver signals the “presence”, or “presence in limited measure”, or “absence” of the behavioral problem. Items are grouped according to psychopathological areas, making the evaluation of the reported data rapid that can easily be done in moments. This makes the CABI particularly suitable for obtaining the firsthand information about the behavior problems of their children-adolescents from the parents/caregivers, preliminary to the visit. Therefore, it provides cues for the detailed data collection during the clinical interview. Moreover, the CABI is a handy tool for epidemiological studies, due to its limited number of items that, although covering almost the entire psychopathology, does not tire out or discourage the responder.

Here, a more extensive validation is reported with normative data of the CABI for a population aged 6 to 17 years along with the preliminary results of the use of the questionnaire in a population of patients to evaluate the ability of the questionnaire to signal clinical problems.

## MATERIAL AND METHODS

### Subjects

#### Normative Study

The study was carried out in the schools of metropolitan area of Cagliari, including 3 satellite towns (Monserrato. Quartu and Selargius) in a balanced distribution in relation to their populations. This roughly reflects the socioeconomic stratification of the area. However, no schools of more peripheral villages and rural areas were included; therefore, a somewhat lower socioeconomic level might be under-represented.

The initial target was to obtain about 800 valid questionnaires, 400 for primary school, 200 each for lower and upper secondary. Therefore, the schools of different degree were chosen in relation to this objective and, in the context of the individual schools, the classes, where the questionnaires were administered, were randomly. However, in the final collection of the filled questionnaires, it was found that about 14% of the questionnaires were returned. There was no possibility to integrate the number of missing questionnaires in order to reach the initial target.

Schools’ councils approved the distribution of the questionnaires, and written informed consent was obtained from the responding parents/caregivers.

The parents/caregivers of 691 students aged 6 to 17 years agreed to respond to the CABI. However, the final available material in the study included 659 questionnaires, since 32 were excluded (missing or double answers in the main psychopathological areas). Data concerning the population subdivided in 3 age groups (according to the 3 different stages of the Italian school: primary, lower and upper secondary), are shown in Table **1**, together with the normative scores.

#### The Instrument

The CABI was developed with reference to each psychopathological area explored, and the items taking into account as much as possible the descriptions stated in the “Diagnostic criteria” of the DSM-IV-TR. The same, a posteriori, are also in agreement with DSM 5. Data on the selection of the initial 110 items proposed and the evaluation of their comprehensibility by parents are given in [1], where validation procedures are also reported.

#### Clinical Study

The CABI was administered to parents/caregivers of outpatients and inpatients of the Unit of Child & Adolescent NeuroPsychiatry at the “A. Cao” Paediatric Hospital in Cagliari. Together with CABI, the Child Behavior CheckList 6-18 (CBCL) [2], was also administered to the parents/caregivers. The number of the pairs of valid questionnaires obtained was 84.

#### Ethical Approval

The study was approved by the Ethics Committee of the Azienda Ospedaliero-Universitaria di Cagliari (University Hospital of Cagliari), and partially supported by grants from the Regione Autonoma Sardegna and the Fondazione Banco di Sardegna.

### Methodology

#### Normative Study

Parents/caregivers completed the CABI mostly at school, while some at home. Both parents were asked to answer the questionnaires together. However, questionnaires compiled by a single parent were accepted as well.

The parents had the choice of keeping their questionnaire anonymous. If, however, during evaluations some problems emerged, if traceable, they would be advised to get free consultation from a neuropsychiatrist or psychologist.

#### Clinical Study

Parents/caregivers completed the CABI in the waiting room before the clinical consultation for out-patient or day hospital patient, or on the first given occasion (usually the first day) in case of hospitalization. Children and adolescents with any type of behavioral/psychiatric disorder were investigated for the evaluation of the CABI. After a whole the K-SADS [3]-supported clinical evaluation, leading to a complete definition of the psychopathological symptoms and of the diagnosis formulation, data were compared with those indicated by the CABI and CBCL.

Since the original scales of the CBCL do not correspond adequately with the clinical definitions, the comparison was made using the more recent DSM-oriented scales [4]. Of these, the “somatic problems” scale was not taken into account, since it does not correspond to clinical disorders, and only requires the completion of other scales such as anxiety and depression.

The comparison was made between the clinical definition of the presence or absence of anxiety, depression, oppositional defiant disorder (ODD), conduct disorder (CD) and attention deficit hyperactive disorder (ADHD), and the scores obtained with CABI and CBCL. The comparison was separately made with both “borderline” (T>65) and with “clinical” (T>70) scores of both the scales. This is in agreement with the criterion used in the evaluation of the CBCL, and gives a more detailed assessment of the capabilities of the questionnaires.

### Analysis of the Data

Scores derived from the answers given in the questionnaires were transferred to an Excel file and elaborated using the SPSS program.

Mean and standard deviation values were calculated in the questionnaires subdivided according to sex and 3 age groups: 6-10, 11-13 and 14-17 years old.

Cronbach alpha was used for the internal consistency.

The extraction method of principal component analysis was used for exploratory factor analysis, followed by the Varimax rotation with Kaiser normalization.

The results of the school children were compared with those suffering from psychopathology in order to evaluate the discriminant capability of the main CABI scales, using the Mann&Whitney test U. Moreover, in the clinical population, the results of the CABI were compared with those of the CBCL (chi^2^ test), and both the questionnaires were compared with the definitive clinical evaluation.

## RESULTS

After excluding the questionnaires with one or more missing data, a final normative sample was obtained regarding 659 children (335 females and 324 males), aged 6-17 years: 387 (204 F, 183 M) attending primary school (6-10 y.o.), 127 (62 F, 65 M) lower secondary school (ages 11-13) and 145 (69 F, 76 M) upper secondary school (ages 14-17).

### Normative Values

Table **1** shows mean and SD values obtained from 659 children of the school and adolescents of 6-17 years old in 22 areas explored by the CABI, divided by the age groups and gender.

As expected, the scores were different in relation to age and gender.

In general, mean values for externalizing disorders were higher in males compared to females, with the highest values for ADHD in the 6-10 y.o. children group, where the scores of males (3.2+2.5) were significantly higher than those of females (2.4+2.4; p=.0014).

On the other hand, the mean scores of internalizing disorders and of eating disorders tended to be slightly higher in females, however; without a significant difference.

Other parameters did not show significant differences either by age or by gender.

The graphical distribution of the data of the school population gave curves with positive skewness and higher frequency of the lowest scores.

### Internal Consistency

In the school population, Cronbach’s test for the whole CABI, all ages and genders included, gives alpha value .833, that qualified as “good”.

### Factor Analysis

Exploratory factor analysis was performed on the questionnaires from the group of 659 school children. Due to the heterogeneity of the psychopathological areas explored, the analysis was focused on items related to the two main disease groups, internalizing and externalizing. Therefore items of anxiety (5-10), depression (19-28), irritability (29-32), ODD (33-37), CD (38-42) and ADHD (43-51) were selected; moreover, items nos.11 (fears) and 16-17 (insecurity) were included.

The analysis showed the best differentiation in 3 factors. Factor 1 included the items from 29 to 50 (irritability, ODD, CD and ADHD) with the exclusion of the items 45 (“He interrupts, disturbing games and others’ conversations”) and 51 (“He gets tired very quickly even when he is playing”).

Factor 2 included the items from 5 to 26 (anxiety with phobias, insecurity, depression) with the exclusion of items 8 (“It is hard for him to be separated or far from his parents”), 25 (“He is often tired or listless; everything exhausts him”), 27 (“He has sometimes said he does not want to live any longer”) and 28 (“He has hurt himself or tried to hurt himself”). The 3^rd^ group included the other items with rather heterogeneous scores.

### Discriminant and Comparative Validation

The data concerning the discriminant validation, that is the capability of the CABI to distinguish the normal subjects from pathological subjects, and the comparative validation, that is the comparison with the validated CBCL instrument, are shown in Table **2**. The comparison was made using not only the first diagnosis, but also the co-morbidities of the clinical sample. Then, for example, when a patient was diagnosed to have a depressive disorder with comorbidity of an anxious disorder, it was evaluated if the pertinent scores of the CABI and of the CBCL were indicative for each one of these comorbidities. This made it possible to obtain a total of 206 comparisons. Besides the scores T>70, in the range of pathology, the scores T>65, including “borderline” condition, were also compared (Table **2**).

As shown in Table **2**, the CABI showed a high concordance with the comprehensive clinical evaluation for depressive symptoms and ODD. Compared to the CBCL, the CABI showed higher agreement percentages with the clinical evaluation concerning either depression, anxiety, ODD. No differences were found in the few cases of CD, while CBCL was better in agreement with the clinical evaluation in few cases of ADHD. However, all the comparisons did not show any significant differences.

The comparison (Mann&Whitney U test) between the school population and the clinical showed a highly statistically significant difference in all the items of depression, ODD, CD, ADHD (for all p<.000) and for 5 of anxiety (p<.000), with no difference in the item number 7, related to anxiety (“He worries about school too much”). This may explain the limited ability of the 6th-item to discriminate between the pathological and normal subjects.

## DISCUSSION

The results obtained from a large school population, representative of three hundred thousand inhabitants of the metropolitan area with a balanced and different socio-economic status, allowed the normative data to be used for the CABI based on the general population, excluding people from rural areas. Through comparison between clinical evaluation and the results of the extensively used instrument CBCL, the reliability of the questionnaire to characterize the subjects in clinical samples was assessed.

All the items for which the comparison was made clearly differentiated normal subjects from the pathological subjects, with the only exception of item 7 (“He worries about school too much”). Furthermore, the results of the CABI with the clinical evaluation, although referring to a limited number of patients, appeared similar and sometimes slightly better that that obtained with the CBCL. These results support the utility and validity of the use of the CABI.

A few other questionnaires were also prepared for collecting the information from the parents for the clinical evaluation of children and adolescents, in order to provide an initial series of data to be further investigated in the clinical interview. The “Strengths and Difficulties Questionnaire” by Goodman [5] only had 25 items, which limited the exploration to few areas. The “Ontario Child Health Study” scale by Boyle *et al*. [6] is a well constructed scale containing 62 items, limited to externalizing and internalizing disorder. The “CASI-4 Parent Checklist” by Gadow & Sprafkin [7], containing 142 items, and the BASC-2 by Reynolds & Kamphaus [8], containing 160 items, are rather long and covered by the copyright.

The CBCL [2] is the first (1990) and the most widely used instrument, with more than 2000 citations in PubMed. However, its administration time is rather long (113 items), which could discourage parents from giving accurate responses. The construct validity of the original syndrome dimensions of the CBCL has been questioned [9], leading to a new grouping of the items in 6 scales [4] coherent with the DSM’s recent definition [10, 11]. The items used in the 6 DSM-oriented scales were only 55 out of 113 administered, with a rather different distribution between 6 scales (ODD 5 items, Anxiety 6, Somatic and ADHD 7, Affective (Depressive) 13, Conduct 17). The CBCL is covered by the copyright and therefore may represent an economic burden.

The CABI contains items mainly based on the diagnostic criteria of the DSM-IV-TR [10] preserved in the DSM-5 [11]. The distribution is more equilibrate (5 Anxiety, 4 Somatic, 10 Depression, 5 ODD, 5 CD and 9 ADHD). Unlike the CBCL, items referring to a specific area were organized together: this helped the parents to more easily understand the specific problems when responding to the questionnaire, providing the clinician rapid and the first-glance evaluation of the subject, without requiring the elaboration of the answers as in the CBCL.

Therefore, CABI appears to be a practical and reliable instrument. Its recommended use is before the clinical evaluation, although it can be used in subsequent phases as a complementary aid for rapid exploration of all the possible problematic areas. An interesting application may be also the comparison of the evaluations of the parents through the CABI with those expressed by their sons-daughters through a self-administered instrument, as done for anxious and depressive symptoms with the scales of SAFA [12] in the preceding paper [1].

For use as a screening instrument, the advantage of lesser number of items compared to the CBCL is remarkable since respondents are generally less motivated and therefore they may not contribute efficiently due to a long questionnaire.

## WHAT IS ALREADY KNOWN

The CABI is a new questionnaire to be filled-out by parents/caregivers, requiring the definition of normative scores for the population of 6-17 years old and a verification of its clinical validity. The CABI is one of the questionnaires for use in child and adolescent psychiatry, both for the preparation or completion of the clinical evaluation, and for screening and epidemiological purpose. Its advantage over similar instruments like the most widely used CBCL is the lower number of items, while exploring virtually all psychopathology.

## WHAT THIS PAPER ADDS

The use of the CABI in a large school population shows its validity, providing normative data for children and adolescents. Moreover, its use as a clinical sample, although still has limitations, suggests clinical reliability of the CABI, being perhaps superior to that of the CBCL.

## Figures and Tables

**Table 1:** Scores obtained in the school population in each of the 22 areas explored by the CABI, subdivided by sex and age groups. The numbers below the name of each area refer to the related items. ADHD scores are reported as divided in the 3 subgroups, and as total. Mean values and (in brackets) standard deviations. F=females, M=males, and n= number of subjects.

		**Somatic 1-4**	**Anxiety 5-10**	**Phobias 11**	**OCD 12-15**	**Insecur- ity 16-17**	**PTSD 18**	**Depres- sion 19-28**	**Irritability 29-32**	**ODD 33-37**	**CD 38-42**	**Impuls 43-45**	**Hyperact 46-48**	**Atten 49-51**	**ADHD tot 43-51**	**Reality 52-55**	**Relation- ships 56-61**	**Enuresis- encopr 62-63**	**Bulimia 64**	**Anorexia 65-67**	**Sex 68-69**	**Substance abuse 70-72**	**School 73-74**	**Bullism victim 75**
6-10 F n = 204	M (SD)	1.2 (1.3)	2.0 (1.7)	0.6 (0.7)	0.4 (0.8)	0.5 (0.7)	0.1 (0.2)	1.0 (1.3)	0.8 (1.2)	0.9 (1.3)	0.1 (0.5)	0,7 (1,4)	0,8 (1,4)	0,8 (1,4)	2.3 (2.4)	0.2 (0.4)	0.2 (0.7)	0.0 (0.1)	0.1 (0.4)	0.2 (0.5)	0.0 (0.1)	0.1 (0.2)	0.1 (0.3)	0.1 (0.3)
6-10 M n = 183	M (SD)	1.0 (1.2)	1.8 (1.5)	0.6 (0.7)	0.4 (0.8)	0.5 (0.7)	0.1 (0.2)	0.9 (1.2)	0.8 (1.3)	1.0 (1.2)	0.2 (0.4)	1,0 (1,5)	1,2 (1,6)	1,0 (1,5)	3.1 (2.5)	0.2 (0.5)	0.3 (0.7)	0.0 (0.2)	0.1 (0.4)	0.2 (0.6)	0.0 (0.2)	0.0 (0.4)	0.3 (0.8)	0.1 (0.3)
11-13 F n = 62	M (SD)	0.6 (0.9)	1.7 (1.6)	0.4 (0.5)	0.4 (1.0)	0.5 (0.9)	0.0 (0.2)	1.0 (0.8)	0.5 (0.8)	0.6 (1.0)	0.1 (0.2)	0,6 (1,1)	0,5 (1,0)	0,7 (1,4)	1.8 (1.7)	0.1 (0.3)	0.2 (0.5)	0.0 (0.1)	0.1 (0.3)	0.3 (0.8)	0.0 (0.1)	0.3 (0.7)	0.1 (0.3)	0.1 (0.2)
11-13 M n = 65	M (SD)	0.8 (0.8)	1.8 (1.9)	0.4 (0.5)	0.7 (1.0)	0.5 (0.8)	0.1 (0.2)	0.9 (1.4)	1.0 (1.4)	0.9 (1.4)	0.2 (0.5)	0,6 (0,8)	0,6 (0,9)	0,7 (1,1)	1.9 (1.7)	0.3 (0.5)	0.6 (1.1)	0.0 (0.1)	0.1 (0.2)	0.1 (0.3)	0.0 (0.1)	0.3 (0.7)	0.4 (0.7)	0.1 (0.3)
14-18 F n = 69	M (SD)	0.7 (0.8)	1.8 (1.7)	0.5 (0.6)	0.3 (0.5)	0.5 (0.6)	0.0 (0.2)	0.9 (1.3)	0.6 (0.8)	0.8 (1.2)	0.2 (0.4)	0,5 (1,1)	0,4 (0,8)	0,8 (1,3)	1.7 (1.6)	0.1 (0.3)	0.3 (0.6)	0.0 (0.1)	0.2 (0.5)	0.4 (0.7)	0.0 (0.1)	0.5 (1.0)	0.4 (0.7)	0.2 (0.5)
14-18 M n = 76	M (SD)	0.9 (1.0)	1.9 (1.5)	0.3 (0.5)	0.9 (1.1)	0.4 (0.6)	0.1 (0.2)	1.0 (1.2)	1.3 (1.3)	1.0 (1.5)	0.2 (0.6)	0,8 (1,1)	0,5 (1,0)	0,6 (1,0)	1.9 (1.7)	0.2 (0.5)	0.4 (0.7)	0.0 (0.1)	0.1 (0.3)	0.3 (0.8)	0.1 (0.2)	0.7 (1.2)	0.4 (0.5)	0.3 (0.5)

**Table 2:** Comparison of the results obtained with the CABI and the CBCL respect to the clinical evaluation.

		CABI T-score ≥ 65	CBCL T-score ≥ 65	CABI T-score ≥ 70	CBCL T-score ≥ 70
Clinical Evaluation	N	n (%)	n (%)	n (%)	n (%)
Depressive symptoms	82	71 (87)	64 (78)	69 (84)	54 (66)
Anxiety symptoms	60	36 (60)	33 (55)	32 (53)	25 (42)
Oppositional Defiant symptoms	26	24 (92)	19 (75)	23 (87)	18 (69)
Conduct symptoms	6	4 (67)	3 (50)	4 (67)	3 (50)
ADHD	6	4 (67)	6 (100)	2 (33)	6 (100)

## LIST OF ABBREVIATIONS

ADHD: = Attention Deficit Hyperactivity Disorder
CABI: = Child and Adolescent Behavior Inventory
CBCL: = Child Behavior Check-List
CD: = Conduct Disorder
DSM-IV-TR: = Diagnostic Statistic Manual-IV-Text Revised
DSM 5: = Diagnostic Statistic Manual 5^th^ edition
F: = female/s
K-SADS-PL: = Kiddie-Schedule for Affective Disorders and Schizophrenia-Present and Lifetime
M: = male/s
ODD: = Oppositional Defiant Disorder
SAFA: = self-administered psychiatric scale for children and adolescents
